# The effect of graphene–poly(methyl methacrylate) fibres on microbial growth

**DOI:** 10.1098/rsfs.2017.0058

**Published:** 2018-04-20

**Authors:** Rupy Kaur Matharu, Harshit Porwal, Lena Ciric, Mohan Edirisinghe

**Affiliations:** 1 Department of Mechanical Engineering, University College London, Torrington Place, London WC1E 7JE, UK; 2 School of Engineering and Materials Science, Queen Mary University of London, Mile End Road, London E1 4NS, UK; 3 Department of Civil, Environmental & Geomatic Engineering, University College London, Chadwick Building, Gower Street, London WC1E 6BT, UK

**Keywords:** graphene, bacterial activity, gyration, nanomaterials, fibres, pressurized gyration

## Abstract

A novel class of ultra-thin fibres, which affect microbial growth, were explored. The microbial properties of poly(methyl methacrylate) fibres containing 2, 4 and 8 wt% of graphene nanoplatelets (GNPs) were studied. GNPs were dispersed in a polymeric solution and processed using pressurized gyration. Electron microscopy was used to characterize GNP and fibre morphology. Scanning electron microscopy revealed the formation of beaded porous fibres. GNP concentration was found to dictate fibre morphology. As the GNP concentration increased, the average fibre diameter increased from 0.75 to 2.71 µm, while fibre porosity decreased. Gram-negative bacteria *Escherichia coli* and *Pseudomonas aeruginosa* were used to investigate the properties of 2, 4 and 8 wt% GNP-loaded fibres. GNP-loaded fibres (0 wt%) were used as the negative control. The fibres were incubated for 24 h with the bacteria; bacterial colony-forming units were enumerated by adopting the colony-counting method. The presence of 2 and 4 wt% GNP-loaded fibres promoted microbial growth, while 8 wt% GNP-loaded fibres showed antimicrobial activity. These results indicate that the minimum inhibitory concentration of GNPs required within a fibre is 8 wt%.

## 1. Introduction

Carbon-based nanomaterials, such as zero-dimensional fullerenes, one-dimensional carbon nanotubes (CNTs), two-dimensional graphene sheets, three-dimensional graphite, single-walled carbon nanohorns, carbon quantum dots, nanodiamonds, graphene oxide (GO) and its derivatives possess unique advantageous properties that have gained considerable attention in a multitude of research fields. Since their inceptive discovery, these materials have been used in materials science and engineering [1], electronics [2], environmental engineering [3] and biomedical engineering [4–7].

Among the bounteous properties carbon-based nanomaterials possess, their effect on microbial growth remains undetermined. It has been well documented that viability for microbial growth, particularly in aquatic systems, is dependent on the carbon content available in the immediate environment [8,9]. Several studies have shown a positive correlation between carbon source metabolism and microbial proliferation, with the carbon source often determining the maximum obtainable cell density [10–12].

The antimicrobial properties of carbon-based materials have also been investigated [13–16]. In 2010, Hu *et al*. [13] first reported the destructive interactions between GO and *Escherichia coli*. Akhavan & Ghaderi [14] further demonstrated the antibacterial activity of GO and reduced GO against both Gram-positive and Gram-negative bacteria. Three distinctive mechanisms have been proposed for the antimicrobial activity of carbon-based materials: direct damage to the microbial membrane, production of oxidative stress and microbial encapsulation/agglomeration.

Graphene nanoplatelets (GNPs) are the most recently discovered carbon-based nanomaterial. GNPs are the two-dimensional counterpart of CNTs and are composed of a single layer of sp^2^ hybridized carbon atoms arranged in a regular hexagonal lattice [17,18]. This cyclic configuration [19] increases the exposed surface area (≈ 2630 m^2^ g^−1^) by a factor of two when compared with single-walled CNTs [20]. Each atom is attached to three neighbouring carbon atoms in the *x*–*y* plane by sigma bonds [21]. The atoms also have a weakly delocalized π-electron cloud that is orientated in the *z*-axis [21]. These electron clouds are responsible for the materials’ superior electrical conductivity, adjustable band gap, room temperature quantum Hall effect [22,23] and the π-plasmon resonance [24]. Owing to the novel nature of this material, very little research on its effect on microbial growth exists. Successful utilization of carbon-based nanomaterials ultimately depends on understanding how they interact with microbes. Identifying the minimum inhibitory concentration would allow for safe exploitation of the material in pertinent applications.

Polymeric fibres featuring biologically active agents show great promise in a broad range of applications, including bioreactors and air and water filtration systems for commercial, industrial and defence applications. Fibrous-bed bioreactors and filtration systems have progressively seen increased utilization over the past two decades due to their favourable technical properties.

Implementation of ultrafine fibres in bioreactors has proved to be an effective method to enhance bacterial fermentation productivity [25–28]. The fibrous matrix provides a renewable surface for bacterial survival and growth while also preventing bacterial cell agglomeration [29], both of which can decrease fermentation efficiency. Fibrous-bed bioreactors offer long-term stability for continuous operation without an observable loss in productivity and thus are highly sought after.

In filtration systems micro- and nanofibres are well known to provide superior filter efficiency by capturing particles and microorganisms efficiently through inertial impaction, interception and convective Brownian diffusion [30]. However, previous literature has demonstrated that a multitude of microorganisms are capable of colonizing modern filtration systems [31–34]. In addition, the organic/inorganic particulates deposited on the fibres post-filtration can facilitate microbial proliferation. This consequently reduces filter efficiency and promotes filter deterioration (bioporation) [35].

Incorporation of biologically active agents into fibres offers a suitable solution to overcome such complications. This can be achieved through several fibre-forming techniques such as melt extrusion, electrospinning and nozzle-free centrifugal spinning [36–41]. However, several limitations of these methods exist, including: the inability to produce large quantities of high-quality fibres, poor cost–yield efficiency, failure to be up-scaled to meet commercial needs and the requirement of high temperatures and large voltages. Pressurized gyration bestows itself as a suitable alternative to existing techniques [42,43]. During this process, a polymer solution is subjected to centrifugal forces in addition to high pressure in a perforated aluminium cylindrical vessel. This encourages the polymer solution to flow through the orifices, thus creating a multitude of jets. The jets undergo elongation due to the forces acting upon them.

The aim of this study is to manufacture GNP-loaded polymeric fibres via pressurized gyration processing; to understand the effect of GNPs on microbial growth; and to determine the minimum inhibitory concentration required in a fibre to obtain an antibacterial effect. *Escherichia coli* and *Pseudomonas aeruginosa* are two of the most common Gram-negative pathogens that cause both nosocomial and community-acquired infections. *Escherichia coli* and *P. aeruginosa* are motile facultative anaerobes. These non-sporulating microorganisms are typically rod-shaped with diameters ranging between 0.5 and 1.0 µm and lengths ranging between 1 and 5 µm. Gram-negative bacteria, in particular, are able to upregulate or obtain genetic elements that code for antibiotic resistance, and are therefore problematic when eradicating them from the environment [44,45]. For these reasons *E. coli* and *P. aeruginosa* were used as model bacteria to evaluate the microbial activity of 0, 2, 4 and 8 wt% GNP fibres.

## 2. Materials and methods

### 2.1. Materials

Poly(methyl methacrylate) (PMMA; *M*_w_ = 120 000 g mol^−1^), chloroform, phosphate-buffered saline (PBS) and Luria Bertani (LB) broth were purchased from Sigma-Aldrich (Gillingham, UK). LB agar was purchased from Invitrogen (Paisley, UK). Grade C-750 GNPs (size ranges from 100 nm to 1–2 µm with an average thickness of 2 nm) were obtained from XG Sciences (Michigan, USA). Circular stainless steel discs (15.5 mm radius, 0.5 mm thick with a 2 mm^2^ grid) were purchased from The Mesh Company Ltd (Warrington, UK). All materials and reagents were used as received.

### 2.2. Graphene nanoplatelet suspension preparation

Chloroform was selected as the carrier solvent for this study. The solutions were prepared in a two-step process. (i) Appropriate quantities of GNPs were added to 10 ml of chloroform to achieve a GNP concentration of 0, 2, 4 and 8 wt% in the final fibres. The GNP suspension was subsequently sonicated (S800, Branson Ultrasonics) for 2 h to achieve a homogeneous dispersion. (ii) Four grams of PMMA was dissolved in 10 ml of chloroform. The solution was stirred on a magnetic stirrer until completely dissolved. Prior to spinning, the GNP suspension was amalgamated with the polymer solution and allowed to stir for 15 min on a magnetic stirrer before being subjected to pressurized gyration. The final polymer concentration was 20% (w/v).

### 2.3. Pressurized gyration

**Figure 1.**
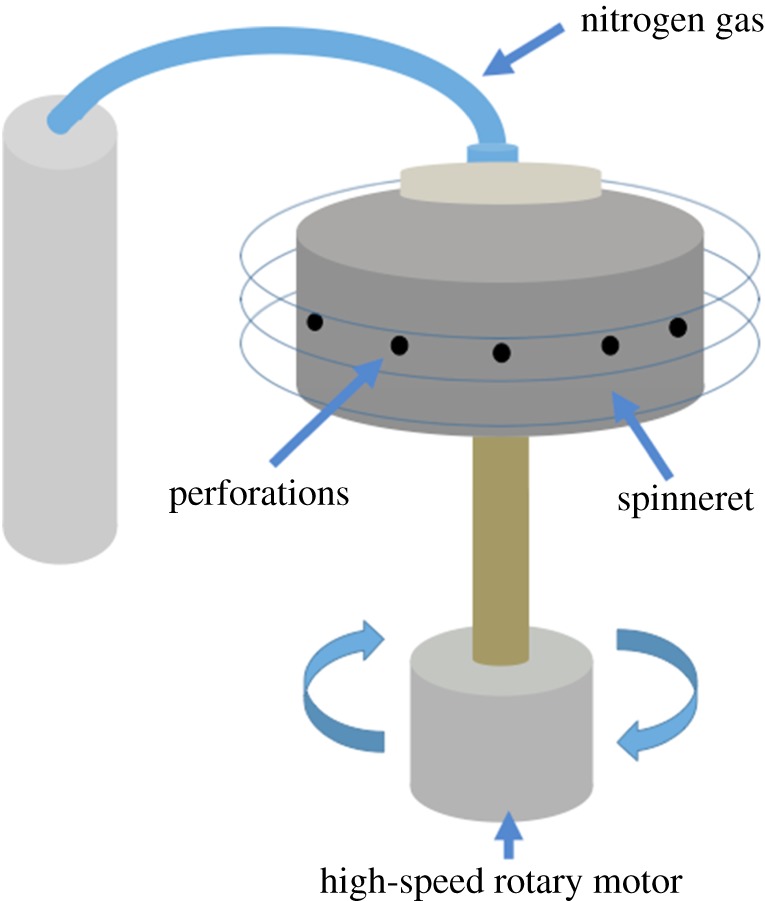
Schematic diagram of the pressurized gyration apparatus used for fibre production. (Online version in colour.)

The solutions were processed for approximately 1 min using pressurized gyration at a rotational speed of 36 000 r.p.m. and an applied pressure of 0.2 MPa. The system consisted of a rotating perforated aluminium cylindrical vessel (30 mm radius and 35 mm tall) fixed to a high-speed rotary motor on one end, and a nitrogen gas supply on the other (figure 1). The vessel had a total of 24 circular perforations, each measuring approximately 0.5 mm in diameter, along the horizontal axis of the vessel. The fibres were collected on sterilized stainless steel mesh discs (31 mm diameter and 0.5 mm thick containing a mesh grid of 2 mm^2^). Pressurized gyration was performed under ambient conditions (19–21°C and 41–46% room humidity). The stainless steel discs coated with 0, 2, 4 and 8 wt% GNP fibres were sterilized under 15 W ultraviolet light for 60 min.

### 2.4. Electron microscopy

#### 2.4.1. Graphene nanoplatelet characterization

GNP characterization was achieved using transmission electron microscopy (TEM). TEM measurements were done using a JEOL JSM-2010 microscope. Samples for TEM were prepared after diluting the GNP suspension and drop casting onto carbon grids.

#### 2.4.2. Fibre morphology

PMMA–GNP composite fibres were analysed using an FEI Inspect-F scanning electron microscope (SEM) after gold sputtering (Emitech sputter coater SC7620) for 90 s. Using high magnification SEM images, average fibre diameter was estimated by measuring the width of approximately 100 fibres. The diameter frequency distribution was also modelled using OriginPro software.

### 2.5. Cell preparation

*Escherichia coli* K12 and *Pseudomonas aeruginosa* NTCC 12903 were used in this study. *Escherichia coli* and *P. aeruginosa* were grown separately in LB broth at 37°C and 150 r.p.m., without the presence of carbon dioxide (Orbital Shaker S150, Stuart) for 3 h and 18 h, respectively. The cells were harvested in their mid-exponential growth phase at an optical density (at 600 nm) of 0.035. The cultures were centrifuged at 4600 r.p.m. for 15 min (accuSpin 3R, Fisher Scientific) to pellet cells. The supernatant was discarded and the remaining cells were washed once with PBS to remove residual macromolecules and other growth constituents. The cells were then resuspended in PBS. The number of cells present in each suspension was established using the colony-counting method.

### 2.6. Microbial studies

The *E. coli* and *P. aeruginosa* cell suspensions made in §2.5 were incubated with sterilized 0, 2, 4 and 8 wt% GNP fibres for 24 h at 37°C and 150 r.p.m., without the presence of carbon dioxide. The number of colony-forming units present in the suspension post-incubation was calculated using the colony-counting method. Bacterial suspensions with and without GNP fibres were incubated for 24 h at 37°C and 150 r.p.m. After incubation, serial 10-fold dilutions were performed on the suspension and then spread onto LB agar plates, which were incubated for 24 h at 31°C. After this time, colony-forming units were counted. Bacterial reduction was calculated as a percentage and compared to the control; 0 wt% GNP-loaded fibres (pure PMMA fibres) were used as the control. All treatments were prepared in triplicate and repeated on at least three separate occasions. A two-tailed *p*-test was performed to assess the statistical significance of the results. The results were considered significant when *p* < 0.05.

## 3. Results and discussion

### 3.1. Electron microscopy

#### 3.1.1. Graphene nanoplatelet morphology

**Figure 2.**
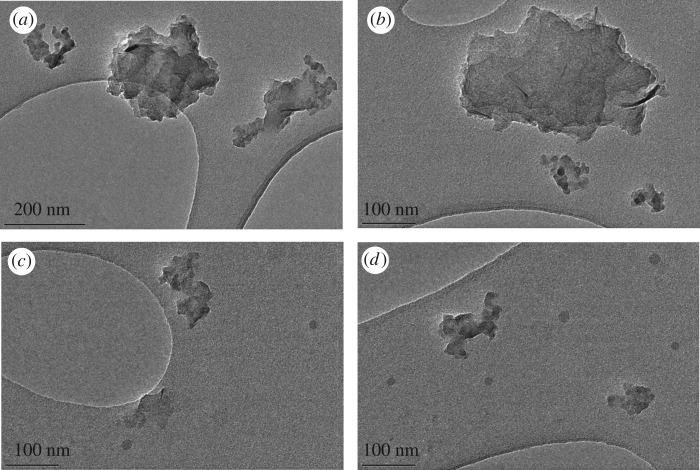
TEM micrographs of the GNPs used in this research.

TEM images of the GNPs were used to gather information on the size, shape and morphological parameters of the individual platelets (figure 2). Analysis revealed the GNPs were relatively flat and two-dimensional. The average width of the individual GNPs was 110 nm (±0.11 nm) and the average length was 170 nm (±0.08 nm). Prior to TEM, the GNPs were sonicated to disperse any aggregates; it is possible that this sonication led to a decrease in GNP dimensions when compared with the specification provided by XG Sciences. The thermogravimetric analysis data supplied by XG Sciences confirm there are traces of amorphous carbon present in the supplied GNPs. This can be seen as grey dots in figure 2*c*,*d*.

#### 3.1.2. Fibre morphology

A PMMA–chloroform polymer–solvent system was opted for during these experiments, based on previous studies having deemed this system suitable for both pressurized gyration and filtration applications [46,47]. The optimal spinning conditions outlined in these papers [46,47] were used for this research. In fact, we can further upstage fibre forming by using pressure coupled infusion gyration [48].

The average fibre diameters and their standard deviations of fibres with varying GNP concentrations are shown in table 1.

**Table 1.** The effect of GNP loading on fibre diameter and distribution.

	fibre diameter	
GNP loading (wt%)	average fibre diameter (μm)	standard deviation (μm)
0	0.75	0.35
2	0.95	0.40
4	0.99	0.56
8	2.71	1.74

**Figure 3.**
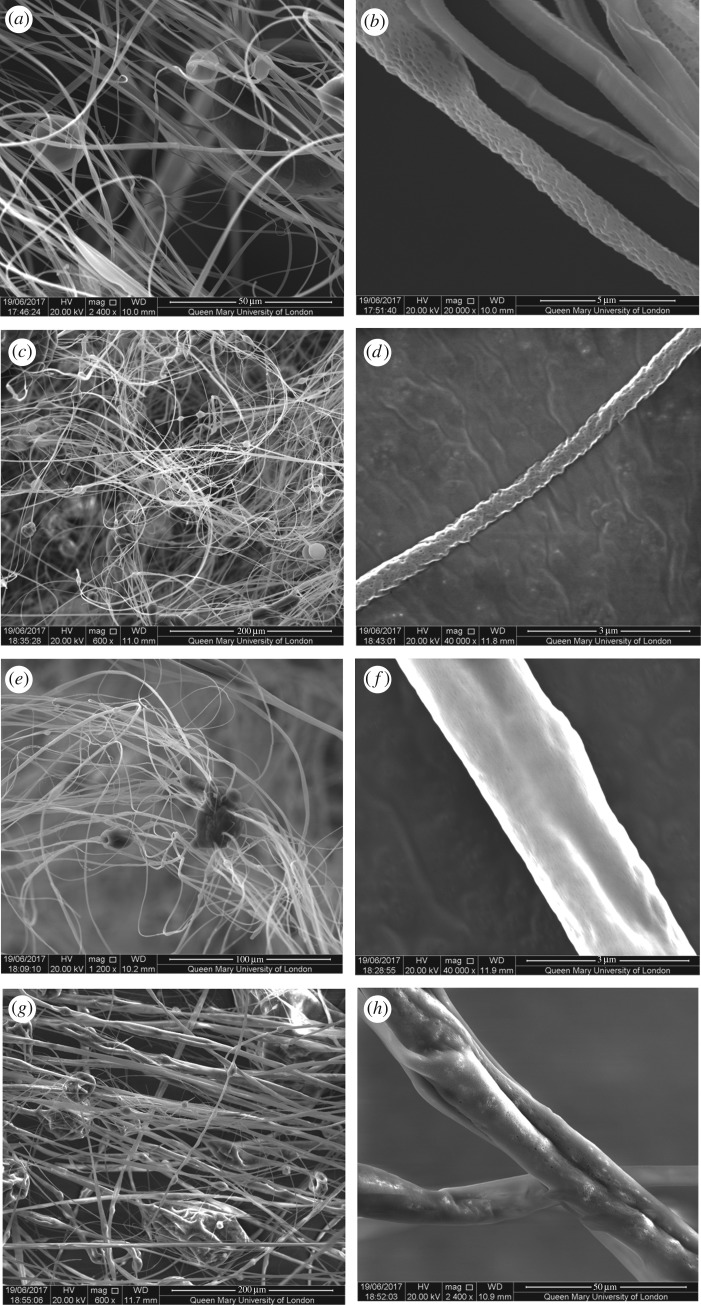
SEM images of the fibres formed using pressurized gyration at 0.2 MPa and 36 000 r.p.m. (*a*) Low magnification SEM image demonstrating fibre morphology of pure PMMA fibres (scale bar = 50 µm); (*b*) high-magnification SEM image illustrating pore morphology of pure PMMA fibres (scale bar = 5 µm); (*c*) low-magnification SEM image of 2 wt% GNP-loaded PMMA fibres (scale bar = 200 µm); (*d*) high-magnification SEM image of 2 wt% GNP-loaded PMMA fibres (scale bar = 3 µm); (*e*) low-magnification SEM image of 4 wt% GNP-loaded PMMA fibres (scale bar = 100 µm); (*f*) high-magnification SEM image illustrating surface topography of 4 wt% GNP-loaded PMMA fibres (scale bar = 3 µm); (*g*) low-magnification SEM image of 8 wt% GNP-loaded PMMA fibres (scale bar = 200 µm); (*h*) high-magnification SEM image of 8 wt% GNP-loaded PMMA fibres (scale bar = 50 µm).

**Figure 4.**
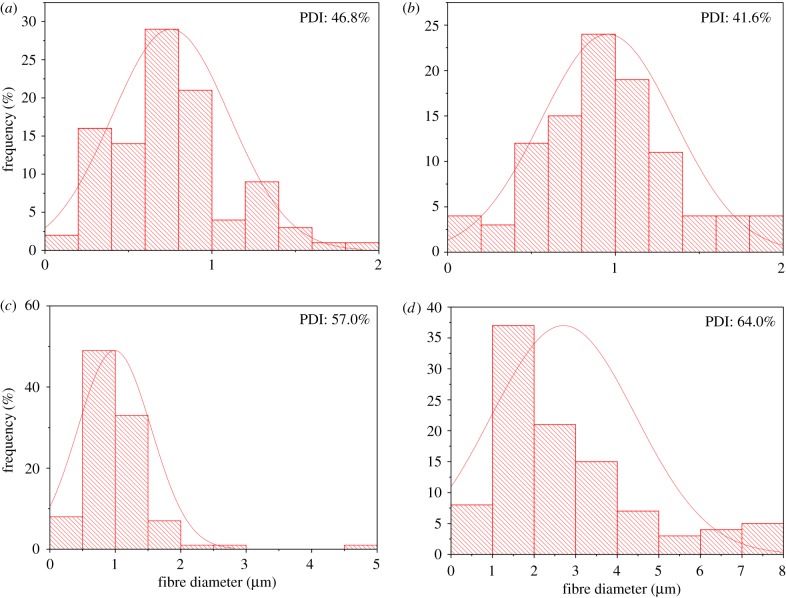
Histograms showing the diameter distribution of the fibres formed; (*a*) pure PMMA fibres; (*b*) 2 wt% GNP-loaded PMMA fibres; (*c*) 4 wt% GNP-loaded PMMA fibres; (*d*) 8 wt% GNP-loaded PMMA fibres. PDI, polydispersity index. (Online version in colour.)

As seen in figure 3*a*,*b*, the 0 wt% GNP-loaded PMMA fibres were continuous, tubular, beaded and highly porous. The successful formation of fibres indicates the intermolecular entanglement and chain overlap in the solution were sufficient to stabilize the polymer jet ejecting from the perforations during pressurized gyration. The average fibre diameter was 0.75 µm, with a minimum and maximum diameter of 0.16 µm and 1.94 µm, respectively. Figure 4*a* shows the fibre diameter distribution, demonstrating a narrow spread, thus allowing for predictable fibre production. The fibres had evenly distributed circular pores on their surface, the formation of which can be explained by the breath figures phenomenon [49]. Being a highly volatile liquid, rapid evaporative cooling of chloroform led to moisture nucleation and water droplet deposition on the surface of the polymer jet. These droplets formed a stable interface between PMMA and water via the adsorption of PMMA, which prevented coalescence [50]. Expansion and submersion of the droplets into the PMMA jet occurred as a result of Marangoni convection and also thermocapillary effects [51,52]. The droplets self-arranged into an ordered array on the solution surface, and evaporation of the solvent and water droplets left pores on the formed fibres.

Fibres loaded with 2 wt% GNPs appeared to share similar morphologies to pure PMMA fibres as they were able to retain their tubular, porous structure (figure 3*c*,*d*). This suggests that, at low concentrations, the GNP does not alter fibre production, as a result of desirable uniform GNP dispersion within the polymer solution. The fibre matrix was beaded and the average fibre diameter obtained was 0.95 µm, with a minimum and maximum diameter of 0.9 µm and 1.96 µm, respectively. The fibre diameter distribution shown in figure 4*b* suggests the diameter distribution is slightly wider when compared with that of pure PMMA fibres.

Increasing the GNP concentration to 4 wt% resulted in an increase in beaded fibres and a decrease in porosity (figure 3*e*,*f*). The rise in bead frequency within the fibre matrix is assumed to be caused by GNP agglomeration. This indicates that at higher GNP loadings, there is a non-homogeneous dispersion of GNPs within the solution, and the solution can therefore be described as being GNP aggregates dispersed within a polymer matrix. The average fibre diameter was found to be 0.99 µm, having diameters ranging from 0.42 µm to 4.79 µm. This average value is similar to that obtained from fibres with low GNP loading, but with a broader fibre diameter distribution (figure 4*c*). The broad range can be explained through two distinct principles. One way in which a wider fibre diameter distribution was obtained is through the difference in the solutions’ rheological properties. The increase in the GNP concentration elicited saturation of the PMMA solution, which stemmed the development of GNP agglomerates. GNP agglomerates consequently caused interference with polymer chain entanglement and altered fibre production. This theory has been corroborated by Weir *et al*. [53], who have demonstrated that GO causes a decrease of interchain entanglements within polymer–GO nanocomposites. Conversely, increased GNP concentration raises the GNP-to-polymer ratio (particle volume fraction), and a larger force is required to overcome the increased surface tension [54]. Nonetheless, functional fibre formation implies that the solution had an adequate surface tension and resistance to withstand the applied centrifugal force and pressure difference to form cone jets at the orifices. The solution successfully overcame shear stresses and was able to elongate into fibres. Low viscosity [55] and high surface tension [54] are presumed as they explain the broad distribution of fibre diameters that were observed. The presence of thick fibres suggests that the surface tension was resistant against the forces acting upon it. The increased surface tension is the result of the increased van der Waals forces between the GNP and PMMA solution [56]. However, at lower viscosities, there is an encouragement for the major polymer jets to form fine fibres, thus leading to the variation in fibre diameter distribution.

The fibre surface appeared to contain indentations instead of pores. Decrease in porosity can be described by several theories such as Henry's law [56]. Chloroform within the solution retains a lower vapour pressure at higher GNP concentrations (and consequently higher surface tensions) [54]. This lower vapour pressure slows evaporation of the solvent, as more heat is required to overcome the van der Waals forces [56], resulting in reduced differences in temperature between the surface and the surrounding atmosphere. This then leads to slower droplet formation, nucleation and rapid PMMA precipitation at the droplet–water interface. The combination of these factors results in the lower porosity.

Increasing the GNP concentration further to 8 wt% resulted in an increase in irregular particles amidst the fibrous structure (figure 3*g*). This phenomenon was due to the failure in achieving the desired GNP dispersion within the PMMA solution. The fibres yielded were thicker and rougher in comparison to fibres obtained with low GNP concentrations. As the processing conditions of each solution remained the same, the change in fibre morphology was regarded as a reflection of the GNP concentration.

The 8 wt% GNP fibres had an average fibre diameter of 2.71 µm and a broad diameter distribution as shown in figure 4*d*. The pores appeared to be isolated and moderately distributed along the fibre, thus indicating low porosity (figure 3*h*).

### 3.2. Microbial studies

*Escherichia coli* and *P. aeruginosa* were incubated in PBS with the fibres for 24 h at 37°C and 150 r.p.m. The microbial activity of the fibres was determined by the colony-counting method as described in §2.6.

**Figure 5.**
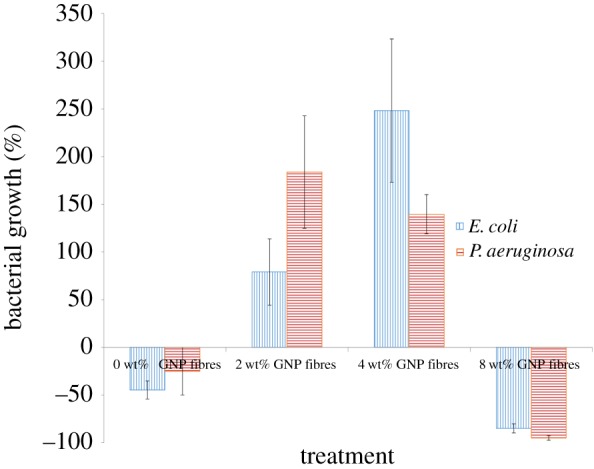
Microbial properties of 0, 2, 4 and 8 wt% GNP-loaded fibres against *E. coli* and *P. aeruginosa*. (Online version in colour.)

As shown in figure 5, 0 wt% GNP (pure PMMA) fibres showed moderate cytotoxicity with an average bacterial reduction of 45 ± 10% and 25 ± 25% compared to the starting culture for *E. coli* and *P. aeruginosa*, respectively. This minor antibacterial effect is likely to be because of the bacterial cell wall damage caused by the hydrophobic interaction between the hydrophobic surface of the PMMA fibres and the hydrophobic domains present on the bacterial cell wall [57,58]. The lack of nutrients present in the PBS and PMMA fibres also played a role in the reduction of bacterial numbers.

GNP fibres (2 wt%) showed promicrobial properties with an average bacterial growth of 79 ± 35% and 184 ± 59% for *E. coli* and *P. aeruginosa*, respectively. Similarly, 4 wt% GNP fibres also demonstrated promicrobial properties, as a 248 ± 75% and 140 ± 21% bacterial increase can be observed in figure 5. It has been well documented in the previous literature that bacterial growth in aquatic systems is dependent on the carbon content available in the environment [8,9]. This suggests that with fibres containing a low concentration of GNPs, the bacterial cells metabolize the GNPs to support microbial growth and cell division. However, additional studies are required to confirm this hypothesis.

When increasing the GNP concentration from 2 wt% to 4 wt%, an increase in *E. coli* and a decrease in *P. aeruginosa* density can be observed. This indicates that the *P. aeruginosa* cells were more sensitive to the increase in GNP concentration compared with the *E. coli* cells. Previous research [59] has demonstrated that *P. aeruginosa* is more susceptible to antimicrobial agents than *E. coli*.

GNP fibres (8 wt%) showed strong antibacterial activity compared with 0, 2 and 4 wt% GNP fibres, having a cell inactivation percentage at 85 ± 5% and 95 ± 2% for *E. coli* and *P. aeruginosa*, respectively. The observed loss of cell viability is considered statistically significant when compared with the control fibres (0 wt% GNPs) as a two-tailed *p*-value of 0.0153 and 0.0474, respectively, for *E. coli* and *P. aeruginosa* was obtained. These results indicate that the minimum inhibitory concentration of GNPs within a fibre is 8 wt%, as at this concentration microbial death occurs.

A multitude of mechanisms can be credited with the antimicrobial activity of these fibres. As the majority of GNPs present are entrapped within the fibres, it is thought that the predominant mechanism of action involves the production of oxidative stress. Graphene-induced oxidative stress is a commonly accepted antimicrobial mechanism, during which the material triggers either the reactive oxygen species-dependent or reactive oxygen species-independent pathway. Activation of either pathway interferes with bacterial metabolism, disrupts essential cellular functions, induces intracellular protein inactivation and causes lipid peroxidation, eventually leading to cellular inactivation, necrosis or apoptosis [60–62].

However, it is also plausible that, at higher GNP concentrations, the mechanical properties of the fibres are weaker and therefore minute quantities of GNP are released into the PBS. The free GNPs suspended in PBS could have caused bacterial cell death by direct damage to the microbial membrane and/or microbial entrapment. Akhavan & Ghaderi [14] were the first to suggest that GNP-initiated antimicrobial activity was caused by the direct interaction between the sharp edges of GNPs and the microbial membrane. In this mechanism, the sharp edges of the GNPs mechanically disrupt the integrity of the microbial membrane and consequently result in the loss of intracellular substances. This phenomenon was later confirmed by several other researchers [14,15,63–65]. This mechanism is the most likely cause of the antimicrobial effect observed in this study. It can clearly be seen that 8 wt% GNP fibres have a very rough surface morphology caused by the GNPs protruding out of the fibres, thus creating sharp edges at the nanoscale. This change in fibre morphology at higher concentrations is most probably the cause of the antimicrobial effects witnessed. The third proposed antimicrobial mechanism, microbial encapsulation/agglomeration, involves the free GNPs wrapping around microbial cells, therefore isolating them from their surrounding environment [63,66,67]. This starves the cells of necessary nutrients required for survival.

All these proposed mechanisms of action are likely to have contributed towards the significant antibacterial activity of the 8 wt% GNP fibres. However, exact mechanisms need further investigation. Promicrobial activity is observed at lower concentrations (2 and 4 wt% GNPs) as the GNPs are trapped within the fibre and the sharp GNP edges do not protrude out and destroy the bacterial cell wall.

## 4. Conclusions

GNP-loaded PMMA fibres were produced using pressurized gyration. The results obtained in this investigation indicated that fibre morphology was dependent on GNP concentration. It was observed that as GNP concentration increased, average fibre diameter increased, average porosity decreased and fibre morphology became increasingly irregular. Average fibre diameter ranged between 0.75 µm and 2.71 µm, for 0, 2, 4 and 8 wt% GNP-loaded fibres.

Understanding the microbial properties of GNP-loaded PMMA fibres is critical for the future application of these emerging carbon-based nanomaterials. The effects of GNPs in PMMA fibres on the growth of *E. coli* and *P. aeruginosa* were compared. Microbial studies revealed that 2 and 4 wt% GNP-loaded fibres showed promicrobial activity, while 8 wt% GNP fibres had antimicrobial activity. These findings suggest that the effects of the fibres on microbial cell growth and division were concentration-dependent. The bacterial growth observed with lower GNP-concentration fibres may be attributed to GNPs serving as a nutrient source for microbial growth. The bacterial cytotoxicity of fibres with a higher GNP concentration may be the result of GNP-induced oxidative stress, as well as membrane destruction and microbial encapsulation. However, further studies will need to be conducted in order to identify an exact mechanism.

## Data accessibility

Data supporting this paper are contained in this paper.
